# CsFEVER and CTKFacts: acquiring Czech data for fact verification

**DOI:** 10.1007/s10579-023-09654-3

**Published:** 2023-05-03

**Authors:** Herbert Ullrich, Jan Drchal, Martin Rýpar, Hana Vincourová, Václav Moravec

**Affiliations:** 1 Artificial Intelligence Center, Faculty of Electrical Engineering, Czech Technical University in Prague, Charles Square 13, 120 00 Prague 2, Czech Republic; 2 Department of Journalism, Faculty of Social Sciences, Charles University, Smetanovo nábřeží 6, 110 01 Prague 1, Czech Republic

**Keywords:** Automated fact-checking, Czech, Document retrieval, Natural language inference, FEVER

## Abstract

In this paper, we examine several methods of acquiring Czech data for automated fact-checking, which is a task commonly modeled as a classification of textual claim veracity w.r.t. a corpus of trusted ground truths. We attempt to collect sets of data in form of a factual claim, evidence within the ground truth corpus, and its veracity label (*supported*, *refuted* or *not enough info*). As a first attempt, we generate a Czech version of the large-scale FEVER dataset built on top of Wikipedia corpus. We take a hybrid approach of machine translation and document alignment; the approach and the tools we provide can be easily applied to other languages. We discuss its weaknesses, propose a future strategy for their mitigation and publish the 127k resulting translations, as well as a version of such dataset reliably applicable for the Natural Language Inference task—the CsFEVER-NLI. Furthermore, we collect a novel dataset of 3,097 claims, which is annotated using the corpus of 2.2 M articles of Czech News Agency. We present an extended dataset annotation methodology based on the FEVER approach, and, as the underlying corpus is proprietary, we also publish a standalone version of the dataset for the task of Natural Language Inference we call CTKFactsNLI. We analyze both acquired datasets for spurious cues—annotation patterns leading to model overfitting. CTKFacts is further examined for inter-annotator agreement, thoroughly cleaned, and a typology of common annotator errors is extracted. Finally, we provide baseline models for all stages of the fact-checking pipeline and publish the NLI datasets, as well as our annotation platform and other experimental data.

## Introduction

In the current highly connected online society, the ever-growing information influx eases the spread of false or misleading news. The omnipresence of fake news motivated formation of fact-checking organizations such as AFP Fact Check,^1^ International Fact-Checking Network,^2^ PolitiFact,^3^ Poynter,^4^ Snopes,^5^ and many others. At the same time, many tools for fake news detection and fact-checking are being developed: ClaimBuster (Hassan et al., 2017), ClaimReview^6^ or CrowdTangle^7^; see Zeng et al. (2021) for more examples. Many of these are based on machine learning technologies aimed at image recognition, speech to text, or Natural Language Processing (NLP). This article deals with the latter, focusing on automated fact-checking (hereinafter also referred to as *fact verification*).

Automated fact verification is a complex NLP task (Thorne & Vlachos, 2018) in which the veracity of a textual *claim* gets evaluated with respect to a ground truth corpus. The output of a fact-checking system gives a classification of the claim—conventionally varying between *supported*, *refuted* and *not enough information* available in corpus. For the *supported* and *refuted* outcomes it further supplies the *evidence*, i.e., a list of documents that explain the verdict. Fact-checking systems typically work in two stages (Thorne et al., 2018a). In the first stage, based on the input *claim*, the document retrieval (DR) module selects the *evidence*. In the second stage, the Natural Language Inference (NLI) module matches the *evidence* with the *claim* and provides the final verdict. Table 1 shows an example of data used to train the fact-checking systems of this type.

**Table 1:** Truncated example from CTKFacts train set

**Claim:** Spojené státy americké hraničís Mexikem	
**EN Translation:** *The United States of America share borders with Mexico*	
**Verdict:** SUPPORTS	
**Evidence 1:** “Mexiko a USA sdílejí 3000 kilometrů dlouhou hranici, kterou ročně překročí tisíce Mexičanů v naději na lepší životní podmínky (...)”	
**EN:** *Mexico and the U.S. share a 3,000-kilometre border, thousands of Mexicans cross each year in hopes of better living conditions (...)*	
**Evidence 2:** “Mexiko také nelibě nese, že Spojené státy stále budují na vzájemné, několik tisíc kilometrů dlouhé hranici zeď, která má zabránit fyzickému ilegálnímu přechodu Mexičanů do USA (...)”	
**EN:** *Mexico is also uncomfortable with the fact that the United States is still building a wall on their mutual, several thousand-mile borders to prevent Mexicans from physically crossing illegally into the U.S. (...)*	

Current state-of-the-art methods applied to the domain of automated fact-checking are typically based on large-scale neural language models (Thorne et al., 2018b), which are notoriously data-hungry. While there is a reasonable number of quality datasets available for high-profile world languages (Zeng et al., 2021), the situation for the most other languages is significantly less favorable. Also, most available large-scale datasets are built on top of Wikipedia  (Aly et al., 2021; Sathe et al., 2020; Schuster et al., 2021; Thorne et al., 2018a). While encyclopedic corpora are convenient for dataset annotation, these are hardly the only eligible sources of the ground truth.

We argue that corpora of verified news articles used as claim verification datasets are a relevant alternative to encyclopedic corpora. Advantages are clear: the amount and detail of information covered by news reports are typically higher. Furthermore, the news articles typically inform on recent events attracting public attention, which also inspire new fake or misleading claims spreading throughout the online space.

On the other hand, news articles address a more varied range of issues and have a more complex structure from the NLP perspective. While encyclopedic texts are typically concise and focused on facts, the style of news articles can vary wildly between different documents or even within a single article. For example, it is common that a report-style article is intertwined with quotations and informative summaries. Also, claim validity might be obscured by complex temporal or personal relationships: a past quotation like *“Janet Reno will become a member of the Cabinet.”* may or may not support the claim *“Janet Reno was the member of the Cabinet.”*^8^ This depends on, firstly, which date we verify the claim validity to, and secondly, who was or what was the competence of the quotation’s author. Note that similar problems are less likely in encyclopedia-based datasets like FEVER (Thorne et al., 2018a).

The contributions of this paper are as follows:

1. FEVER ** localization scheme (and** CsFEVER **case study):** We propose an experimental localization scheme of the large-scale FEVER  (Thorne et al., 2018a) fact-checking dataset, utilizing the public MediaWiki interlingual document alignment of Wikipedia articles and a MT-based claim transduction. We publish our procedure to be used for other languages, and analyze its pitfalls. We observe the types of translation loss on our CsFEVER dataset obtained through this procedure and approximate their frequency. We denote the original English FEVER as EnFEVER in the following sections to distinguish various language mutations.
2. **CTKFacts:** we introduce a new Czech fact-checking dataset manually annotated on top of approximately two million Czech News Agency^9^ news reports from 2000 to 2020. Inspired by FEVER, we provide an updated and extended annotation methodology aimed at annotations of news corpora, and we also make available an open-source annotation platform. The *claim generation* as well as *claim labeling* is centered around limited knowledge context (denoted *dictionary* in Thorne et al. (2018a)), which is trivial to construct for hyperlinked textual corpora such as Wikipedia. We present a novel approach based on document retrieval and clustering. The method automatically generates dictionaries, which are composed of both relevant and semantically diverse documents, and does not depend on any inter-document linking. We present a manually cleaned set of 3k labeled claims from our annotations with 63% Fleiss’ $\kappa$-agreement, backed by evidence from the Czech News Agency archive (CTK corpus). We also publish its standalone (evidence-included) version we call CTKFactsNLI .
3. We provide a detailed analysis of the CTKFacts dataset, including the empirical (based on manual conflict resolution) and *spurious cue analysis*, where the latter detects annotation patterns possibly leading to overfitting of the NLP models. For comparison, we analyze the spurious cues of CsFEVER as well. We construct an annotation cleaning scheme that involves both manual and semi-automated procedures, and we use it to refine the final version of the CTKacts dataset. We also provide classification and discussion of common annotation errors for future improvements of the annotation methodology.
4. We present baseline models for both DR and NLI stages as well as for the full fact-checking pipeline.
5. We publicly release the CTKFacts dataset as well as the experimental CsFEVER data, used source code and the baseline models. Due to the weaknesses of the CsFEVER dataset revealed in Sect. 3 and to the licensing of the ground-truth corpus underlying CTKFacts, we also publish their NLI versions CsFEVER-NLI and CTKFactsNLI that can be used on their own and do not suffer from the transduction noise. Data, tools, and models are available under the CC BY-SA 3.0 license.

This article is structured as follows: in Sect. 2, we give an overview of the related work. Section 3 describes our experimental method to localize the EnFEVER dataset using the MediaWiki alignment. We generate the Czech language CsFEVER dataset with it and analyze its validity. In Sect. 4, we introduce the novel CTKFacts dataset. We describe its annotation methodology, data cleaning, and postprocessing, as well as analysis of the inter-annotator agreement. Section 5 analyzes spurious cues for both CsFEVER and CTKFacts. In Sect. 6, we present the baseline models. Section 7 concludes with an overall discussion of the results and with remarks for future research.

## Related work

This section describes datasets and models related to the task of automated fact-checking of textual claims. More general overview of the state-of-the-art can be found in Zeng et al. (2021) or Murayama (2021).

Emergent (Ferreira & Vlachos, 2016) dataset is based on news; it contains 300 claims and 2k+ articles, however, it is limited to headlines. Due to the dataset size, only simple models classifying to three classes (*for*, *against*, and *observing*) are presented. Described models are fed BoW vectors and feature-engineered attributes.

Wang in Wang (2017) presents another dataset of 12k+ claims, working with 5 classes (*pants-fire*, *false*, *barely-true*, *half-true*, *mostly-true*, and *true*). Each verdict includes a justification. However, evidence sources are missing. The models presented in the paper are claim-only, i.e., they deal with surface-level linguistic cues only. The author further experiments with speaker-related meta-data.

Fact Extraction and VERification (FEVER) (Thorne et al., 2018a) is a large dataset of 185k+ claims covering the overall fact-checking pipeline. It is based on lead sections (hereinafter referred to as “abstracts”) of approximatelly 50k most visited pages of English Wikipedia. Authors present complex annotation methodology that involves two stages: the *claim generation* in which annotators firstly create a true *initial claim* supported by a random Wikipedia source article with context extended by the *dictionary* constructed from pages linked from the source article. The *initial claim* is further *mutated* by rephrasing, negating and other operations. The task of the second *claim labeling* stage is to provide the *evidence* as well as give the final verdict: SUPPORTS, REFUTES or NEI, where the latter stands for the “not enough information” label. Fact Extraction and VERification Over Unstructured and Structured information (FEVEROUS) (Aly et al., 2021) adds 87k+ claims including evidence based on Wikipedia table cells. The size of FEVER data facilitates modern deep learning NLP methods. The FEVER authors host annual workshops involving competitions, with results described in Thorne et al. (2018b) and Thorne et al. (2019).

MultiFC (Augenstein et al., 2019) is a 34k+ claim dataset sourcing its claims from 26 fact checking sites. The evidence documents are retrieved via Google Search API as the ten highest-ranking results. This approach significantly deviates from the FEVER-like datasets as the ground-truth is not limited by a closed-world corpus, which limits the trustworthiness of the retrieved evidence. Also, similar data cannot be utilized to train the DR models.

WikiFactCheck-English (Sathe et al., 2020) is another recent Wikipedia-based large dataset of 124k+ claims and further 34k+ ones including claims refuted by the same evidence. The claims are accompanied by *context*. The evidence is based on Wikipedia articles as well as on the linked documents.

Considering other than English fact-checking datasets, the situation is less favorable. Recently, Gupta and Srikumar (2021) released a multilingual (25 languages) dataset of 31k+ claims annotated by seven veracity classes. Similarly to the MultiFC, evidence is retrieved via Google Search API. The experiments with the multilingual Bert  (Devlin et al., 2019) model show that the gain from including the evidence is rather limited when compared to claim-only models. FakeCovid (Shahi & Nandini, 2020) is a multilingual (40 languages) dataset of 5k+ news articles. The dataset focuses strictly on the COVID-19 topic. Also, it does not supply evidence in a raw form—human fact-checker argumentation is provided instead. Kazemi et al. (2021) released two multilingual (5 languages) datasets, these are, however, aimed at *claim detection* (5k+ examples) and *claim matching* (2k+ claim pairs).

In the Czech locale, the most significant machine-learnable dataset is the Demagog dataset (Přibáň et al., 2019) based on the fact-checks of the Demagog^10^ organisation. The dataset contains 9k+ claims in Czech (and 15k+ in Slovak and Polish) labeled with a veracity verdict and speaker-related metadata, such as name and political affiliation. The verdict justification is given in natural language, often providing links from social networks, government-operated webpages, etc. While the metadata is appropriate for statistical analyses, the justification does not come from a closed knowledge base that could be used in an automated scheme.

The work most related to ours was presented by the authors of Binau and Schulte (2020); Nørregaard and Derczynski (2021), who published a Danish version of EnFEVER called DanFEVER. Unlike our CsFEVER dataset, DanFEVER was annotated by humans. Given the limited number of annotators, it includes significantly fewer claims than EnFEVER (6k+ as opposed to 185k+).

## CsFEVER

In this section, we introduce a developmental CsFEVER dataset intended as a Czech localization of the large-scale English EnFEVER dataset. It consists of claims and veracity labels justified with pointers to data within the Czech Wikipedia dump.

A typical approach to automatically build such a dataset from the En data would be to employ machine translation (MT) methods for both claims and Wikipedia articles.

While MT methods are recently reaching maturity (Dabre et al., 2020; Popel et al., 2020), the problem lies in the high computational complexity of such translation. While using the state-of-the-art MT methods to translate the claims (2.2 M words) is a feasible way of acquiring data, the translation of all Wikipedia articles is a much costlier task, as only their abstracts have a total of 513 M words corresponding to 452k pages (measuring the June 2017 dump used in Thorne et al., 2018a).

However, in NLP research, Wikipedia localizations are often considered a *comparable corpus* (Althobaiti, 2022; Chu et al., 2014; Fišer & Sagot, 2015; Mohammadi & GhasemAghaee, 2010; Štromajerová et al., 2016), that is, a corpus of texts that share a domain and properties. Furthermore, partial alignment is often revealed between Wikipedia locales, either on the level of article titles (Fišer & Sagot, 2015), or specific sentences (Štromajerová et al., 2016)—much like in *parallel* corpora. We hypothesize there may be a sufficient document-level alignment between Czech and English Wikipedia abstracts that were used to annotate the EnFEVER dataset, as in both languages the abstracts are used to summarize basic facts about the same real-world entity.

In order to validate this hypothesis, and to obtain experimental large-scale data for our task, we proceed to localize the EnFEVER dataset using such an alignment derived from the Wikipedia interlanguage linking available on MediaWiki.^11^ In the following sections, we discuss the output quality and information loss, and we outline possible uses of the resulting dataset.

### Method

Our approach to generating CsFEVER from the openly available EnFEVER dataset can be summarized by the following steps:

1. Fix a version of Wikipedia dump in the target language to be the verified corpus.
2. Map each Wikipedia article referred in the evidence sets to a corresponding localized article using MediaWiki API.^12^ If no localization is available for an article, remove all *evidence sets* in which it occurs.
3. Remove all SUPPORTS and REFUTES data points having empty evidence.
4. Apply MT method of choice to all claims.
5. Re-split the dataset to train, dev, and test so that the dev and test veracity labels are balanced.

Before we explore the data, let us discuss the caveats of the scheme itself. Firstly, the evidence sets are not guaranteed to be exhaustive—no human annotations in the target language were made to detect whether there are new ways of verifying the claims using the target version of Wikipedia (in fact, this does not hold for EnFEVER either, as its evidence-recall was estimated to be 72.36% (Thorne et al., 2018a)).

Secondly, even if our document-alignment hypothesis is valid on the level of abstracts, sentence-level alignment is not guaranteed. Its absence invalidates the EnFEVER evidence format, where evidence is an array of Wikipedia *sentence* identifiers. The problem could, however, be addressed by altering the evidence *granularity* of the dataset, i.e., using whole documents to prove or refute the claim, rather than sentences. Recent research on long-input processing language models (Beltagy et al., 2020; Kitaev et al., 2020; Xiong et al., 2021) is likely to make this simplification less significant.

Lastly, the step 5. might allow a slight leakage of information between the splits—while it is guaranteed that no claim appears in two splits simultaneously, two claims extracted from the same Wikipedia article may—however, such claims are typically its different *mutations*, independent of each other’s veracity. This cannot be fixed using publicly available data, if we are aiming for balanced dev , test splits.

Another notable risk of the arbitrary splitting is that of data leakage between localizations—evaluating multilingual models trained on EnFEVER train set will be biased, as it may contain English counterparts of dev and test sets established in step 5. We therefore also encourage maintaining the original EnFEVER splitting, trading off the label balance, in future multilingual experiments.

### Results

Following our scheme from Sect. 3.1, we used the June 2020 Czech Wikipedia dump parsed into a database of plain text articles using the wikiextractor^13^ package and only kept their abstracts. Our resulting Czech Wikipedia corpus has a total of 453k documents, consisting of 1.9 M sentences, 365 M words (including titles).^14^

In order to translate the claims, we have empirically tested three available state-of-the-art English–Czech machine translation engines (data not shown here). Namely, these were: Google Cloud Translation API,^15^ CUBBITT (Popel et al., 2020) and DeepL.^16^ As of March 17th 2021 we observed DeepL to give the best results. Most importantly, it turned out to be robust w.r.t. homographs and faithful to the conventional translation of named entities (such as movie titles, which are very common amongst the 50k most popular Wikipedia articles used in Thorne et al. (2018a)).

Finally, during the localization process, we have been able to locate Czech versions of 6578 out of 12,633 Wikipedia articles needed to infer the veracity of all EnFEVER claims. Omitting the evidence sets that are not fully contained by the Czech Wikipedia and omitting SUPPORTS/REFUTES claims with empty evidence, we arrive to 127,328 claims that can hypothetically be fully (dis-)proven in at least one way using the Czech Wikipedia abstracts corpus, which is 69% of the total 185,445 EnFEVER claims. 24,542 of the claims have more than one listed set of evidence, 15,628 evidence sets are multi-hop, featuring more than one Czech Wikipedia article.

We release the resulting dataset publicly in the HuggingFace datasets repository.^17^ In Table 2 we show the dataset class distribution. It is roughly proportional to that of EnFEVER. Similarly to Thorne et al. (2018a), we have opted for label-balanced dev and test splits, in order to ease evaluation of biased predictors.

**Table 2:** Label distribution in CsFEVER dataset as oposed to the EnFEVER

Split	CsFEVER			EnFEVER		
	SUPPORTS	REFUTES	NEI	SUPPORTS	REFUTES	NEI
Train	53,542	18,149	35,639	80,035	29,775	35,639
Dev	3333	3333	3333	6666	6666	6666
Test	3333	3333	3333	6666	6666	6666

The test split of EnFEVER is not public

#### Validity

In order to validate our hypothesis that the Czech Wikipedia abstracts support and refute the same claims as their English counterparts, we have sampled 1% (1257) verifiable claim-evidence pairs from the CsFEVER dataset and annotated their validity.

**Fig. 1:**
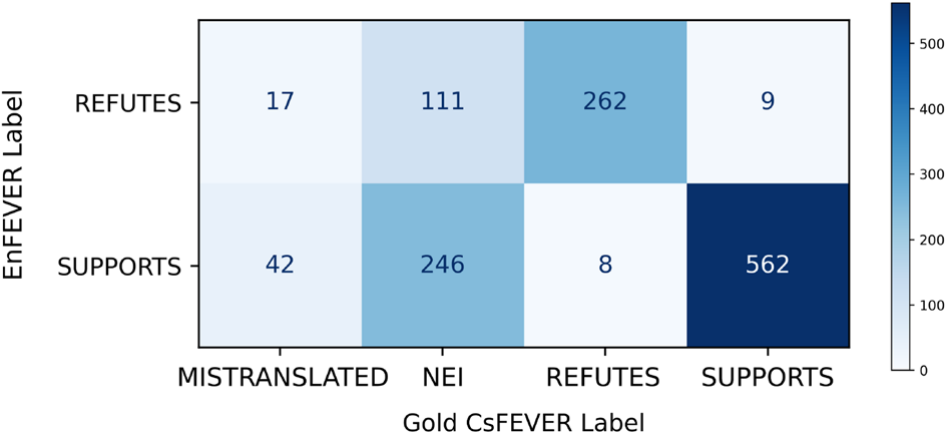
Confusion matrix of the CsFEVER localization scheme

Overall, we have measured a 66% transduction precision with a confusion distribution visualised in Fig. 1—28% of our CsFEVERsample pairs were invalid due to NOT ENOUGH INFO in the proposed Czech Wikipedia abstracts, 5% sample claims were invalidated by an inadequate translation. This number neglects the EnFEVER NEI data points, which were not considered in this measurement and assumed to also not be verifiable using the Czech Wikipedia corpus. However, there is no guarantee that an additional piece of information cannot appear in the new underlying corpus, making a NEI claim verifiable.

We, therefore, claim that the localization method, while yielding mostly valid datapoints, needs a further refinement, and the CsFEVERas-is is noisy and mostly appropriate for experimental benchmarking of model recall in the document-level retrieval task. In Sect. 4.4 we proceed to use this data for training Czech retrieval models for the task of *dictionary generation* vital for our CTKFacts annotations. With caution, it may also be used for NLI experiments.

We conclude that while the large scale of the obtained data may find its use, a collection of novel Czech-native dataset is desirable for finer tasks, and we proceed to annotate a CTKFacts dataset for our specific application case. As Fig. 1 shows a common problem with NEI mislabeling, the dataset could also be further cleaned by a well-performing NLI model at an appropriate level of confidence.

### CsFEVER-NLI

Alternative way to look at the EnFEVER data is to view them as *context*-*query* pairs, where *query* is a claim, and *context* is a concatenation of the full texts of its evidence. This was examined for the NLI task in Nie et al. (2019), and released as the FEVER-NLI dataset. Where no context was given (NEI datapoints without evidence), the authors uniformly sampled 3–5 sentences from the top-ranked Wikipedia abstract according to their retrieval model.

This interpretation of the EnFEVER data reduces the size of Wikipedia content that needs to be translated alongside the claims to 15 M words at the cost of the ability to use the data for retrieval tasks. Therefore, we also publish a dataset we call CsFEVER-NLI that was generated independently on the scheme from Sect. 3.1 by directly translating 228k FEVER-NLI pairs published in Nie et al. (2019) using DeepL. We conclude that by only using the relevant parts of English Wikipedia and translating these, we mitigate most of the problems found in Sect. 3.2.1 and provide a solid dataset for the NLI task on the fact-checking pipeline.

## CTKFacts

In this section, we address collection and analysis of the CTKFacts dataset - our novel dataset for fact verification in Czech. The overall approach to the annotation is based on FEVER  (Thorne et al., 2018a). Unlike other FEVER-inspired datasets (Aly et al., 2021; Nørregaard & Derczynski, 2021; Schuster et al., 2021) which deal with corpora of encyclopedic language style, CTKFacts uses a ground truth corpus extracted from an archive of press agency articles.

As the CTK archive is proprietary and kept as a trade secret, the full domain of all possible evidence may not be disclosed. Nevertheless, we provide public access to the derived NLI version of the CTKFacts dataset we call CTKFactsNLI. CTKFactsNLI is described in Sect. 4.8.

### CTK corpus

For the ground truth corpus, we have obtained a proprietary archive of the Czech News Agency,^18^ also referred to as CTK, which is a public service press agency providing news reports and data in Czech to subscribed news organizations. Due to the character of the service—that is, providing raw reports that are yet to be interpreted by the commercial media—we hypothesize such corpus suffers from significantly less noise in form of sensational headlines, political bias, etc.

Using news corpus as a ground-truth database might be (rightfully) considered controversial. We stress that it is important to select only highly reliable sources for this purpose. Specifically, in the Czech media environment, the CTK is known to keep the high standard of news verification.^19^

The full extent of data provided to our research is 3.3 M news reports published between 1 January 2000 and 6 March 2019. We reduce this number by neglecting redundancies and articles formed around tables (e.g., sport results or stock prices).

Ultimately, we arrive to a corpus of 2 M articles with a total of 13 M paragraphs (35 M sentences), 690 M words. Hereinafter, we refer to it as to the *CTK corpus*, and it is to be used as the verified text database for our annotation experiments.

### Paragraph-level documents

The FEVER shared task proposed a *two-level* retrieval model: first, a set of *documents* (i.e., Wikipedia abstracts) is retrieved. These are then fed to the *sentence retrieval* system which provides the evidence on the sentence level.

This two-stage approach, however, does not match properties of the news corpora—in most cases, the news sentences are significantly less *self-contained* than those of encyclopedic abstract, which disqualifies the sentence-level granularity.

On the other hand, the news articles tend to be too long for many of the state-of-the-art *document retrieval* methods. FEVER addresses a similar issue by trimming the articles to their short abstracts only. Such a trimming cannot be easily applied to our data, as the news reports come often without abstracts or summaries and scatter the information across all their length.

In order to achieve a reasonable document length, as well as to make use of all the information available in our corpus, we opt to work with our full data on the *paragraph* level of granularity, using a single-stage retrieval. From this point onwards, we refer to the CTK paragraphs also as to the *documents*.

We store meta-data for each paragraph, identifying the article it comes from, its order^20^ and a timestamp of publication.

### Source document preselection

In FEVER, every claim is derived from a random sentence of a Wikipedia article abstract sampled from the fifty thousand most popular articles (Thorne et al., 2018a).

With the news report archive in its place, the approach does not work well, as most paragraphs do not contain any check-worthy information. In our case, we were forced to include an extra manual preselection task (denoted $\textsf {T}_{\textsf {0}}$, see Sect. 4.5) to deal with this problem.

### *Dictionary* generation

In EnFEVER *Claim Extraction* as well as in the annotation of DanFEVER  (Binau & Schulte, 2020), the annotator is provided with a source Wikipedia abstract and a *dictionary* composed of the abstracts of pages *hyperlinked* from the source. The aim of such dictionary is to 1) introduce more information on entities covered by the source, 2) extend the context in which the new claim is extracted in order to establish more complex relations to other entities.

With the exception of the *claim mutation* task (see below), annotators are instructed to disregard their own world knowledge. The *dictionary* is essential to ensure that the annotators limit themselves to the facts (dis-)provable using the corpus while still having access to higher-level, more interesting entity relations.

As the CTK corpus (and news corpora in general) does not follow any rules for internal linking, it becomes a significant challenge to gather reasonable dictionaries. The aim is to select a relatively limited set of documents to avoid overwhelming the annotators. These documents should be highly relevant to the given *knowledge query*^21^ while covering as diverse topics as possible at the same time to allow complex relations between entities.

Our approach to generating dictionaries combines NER-augmented keyword-based document retrieval method and a semantic search followed by clustering to promote diversity.

The keyword-based search uses the TF-IDF DrQA  (Chen et al., 2017) document retrieval method being a designated baseline for the EnFEVER  (Thorne et al., 2018a). Our approach makes multiple calls to the DrQA, successively representing the query *q* by all possible pairs of *named entities* extracted from the *q*. As an example consider the query *q* = “Both Obama and Biden visited Germany.”: *N* = “Obama”, “Biden”, “Germany” is the extracted set of top-level named entities. DrQA is then called $$\left( {\begin{array}{c}|N|\\ 2\end{array}}\right) = 3$$ times for the keyword queries *q*_1_ = “Obama, Biden”, *q*_2_ = “Obama, Germany”, *q*_3_ = “Biden, Germany”.

Czech Named Entity Recognition is handled by the model of Straková et al. (2019). In the end, we select at most $n_\text {KW}$ (we use $n_\text {KW} = 4$) documents having the highest score for the dictionary.

This iterative approach aims to select documents describing mutual relations between pairs of NERs. It is also a way to promote diversity between the dictionary documents. Our initial experiments with a naïve method of simply retrieving documents based on the original query *q* (or simple queries constructed from all NERs in *N*) were unsuccessful as journalists often rephrase, and the background knowledge can be found in multiple articles. Hence, the naïve approach often reduces to search for these rephrased but redundant textual segments.

The second part of the *dictionary* is constructed by means of semantic document retrieval. We use the M-Bert  (Devlin et al., 2019) model finetuned on CsFEVER (see Sect. 6.1), which initially retrieves rather large set of $n_\text {PRE} = 1024$ top ranking documents for the query *q*. In the next step, we cluster the $n_\text {PRE}$ documents based on their [CLS] embeddings using *k*-means. Each of the *k* ($k=2$ in our case) clusters then represents a semantically diverse set of documents (paragraphs) $P_i$ for $i \in \{1, \ldots , k\}$. Finally, we cyclically iterate through the clusters, always extracting a single document $p \in P_i$ closest to *q* by means of the cosine similarity, until the target number of $n_\text {SEM}$ (we used $n_\text {SEM} = 4$) documents is reached. The final dictionary is then a union of $n_\text {KW}$ and $n_\text {SEM}$ documents selected by both described methods.

During all steps of dictionary construction, we make sure that all the retrieved documents have an older timestamp than the source. Simply put, to each query, we assign a date of its formulation, and only verify it using the news reports published *to that date*. The combination of the keyword and semantic search, as well as the meta-parameters involved, are a result of empirical experiments. They are intended to provide a minimum neccessary context on the key actors of the claim and its nearest semantical neighbourhood.

In the following text, we denote a dictionary computed for a query *q* as *d*(*q*). In the annotation tasks, it is often desirable to combine dictionaries of two different queries (claim and its source document) or to include the source paragraph itself. For clarity, we use the term *knowledge scope* to refer to such entire body of information.

### Annotation workflow

The overall workflow is depicted in 2 and described in the following list:

**Fig. 2:**
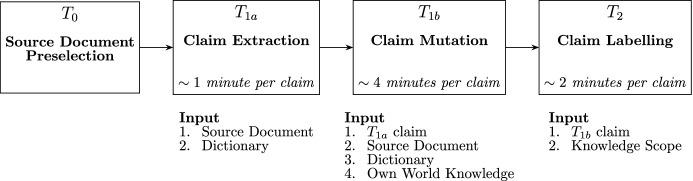
CTKFacts annotation tasks

1. **Source document preselection** ($\textsf {T}_{\textsf {0}}$) is the preliminary annotation step as described in Sect. 4.3, managed by the authors of this paper.
2. **Claim extraction** ($$\textsf {T}_{\textsf {1a}}$$):
  The system samples random paragraph *p* from the set of paragraphs preselected in the $\textsf {T}_{\textsf {0}}$ stage.
  The system generates a dictionary *d*(*p*), querying for the paragraph *p* and its publication timestamp (see Sect. 4.4).
  Annotator *A* is provided the *knowledge scope* $K = \{p\}\cup d(p)$.
  *A* is further allowed to augment *K* by other paragraphs published in the same article as some paragraph already in *K*, in case the provided knowledge needs reinforcement.
  *A* outputs a simple true *initial claim* *c* supported by *K* while disregarding their own world knowledge.
3. **Claim mutation** ($\textsf {T}_{\textsf {1b}}$): The claim *c* is fed back to *A*, who outputs a set of claim mutations: $M=\{m_1,\ldots m_n\}$. These involve the mutation types defined in Thorne et al. (2018a): *rephrase*, *negate*, *substitute similar*, *substitute dissimilar*, *generalize*, and *specify*. We use the term *final claim* interchangeably with the *claim mutation* in the following paragraphs. This is the only stage where *A* can employ own world knowledge, although annotators are advised to preferably introduce knowledge that is likely to be covered in the corpus. To catch up with the additional knowledge introduced by *A*, the system precomputes dictionaries $d(m_1),\ldots d(m_n)$.
4. **Claim labeling** ($$\textsf {T}_{\textsf {2}}$$):
  The annotation environment randomly samples a *final claim* *m* and presents it to annotator $A^\prime$ with a knowledge scope *K* containing the original source paragraph *p*, its $\textsf {T}_{\textsf {1a}}$ dictionary *d*(*p*), as well as the additional dictionary *d*(*m*) retrieved for *m* in $\textsf {T}_{\textsf {1b}}$. The order of *K* is randomized (except for *p* which is always first) not to bias the time-constrained $A^\prime$.
  $A^\prime$ is further allowed to augment *K* by other paragraphs published in the same article as some paragraph already in *K*, in case the provided knowledge needs reinforcement
  $A^\prime$ is asked to spend $\le 3$ minutes looking for minimum evidence sets $E^{m}_1,\ldots ,E^{m}_n$ sufficient to infer the veracity label which is expected to be the same for each set.
  If none found, $A^\prime$ may also label the *m* as NEI.

Note that FEVER defines two subtasks only: *Claim Generation* and *Claim Labeling*. *The Claim Generation* corresponds to our $\textsf {T}_{\textsf {1}}$, while *the Claim Labeling* is covered by $\textsf {T}_{\textsf {2}}$.

#### Annotation platform

Due to notable differences in experiment design, we have built our own annotation platform, rather than reusing that of Thorne et al. (2018a). The annotations were collected using a custom-built web interface. Our implementation of the interface and backend for the annotation workflow described in Sect. 4.5 is distributed under the MIT license and may be inspected online.^22^ We provide further information on our annotation platform in Appendix 1.

#### Annotators

Apart from $\textsf {T}_{\textsf {0}}$, the annotation tasks were assigned to groups of bachelor and master students of Journalism from the Faculty of Social Sciences at the Charles University in Prague. We have engaged a total of 163 participants who have signed themselves for courses in *AI Journalism* and *AI Ethics* during the academic year 2020/2021. We used the resulting data, trained models and the annotation experiment itself to introduce various NLP mechanisms, as well as to obtain valuable feedback on the task feasibility and pitfalls.

The annotations were made in several *waves*—instances of the annotation experiment performed with different groups of students. This design allowed us to adjust the tasks, fullfilment quotas and the interface after each wave, iteratively removing the design flaws.

#### Cross-annotations

In the annotation labeling task, we advised the annotators to spend no more than 2–3 min finding as many evidence sets as possible within Wikipedia, so that the dataset can later be considered almost *exhaustive* (Thorne et al., 2018a). With our CTK corpus, the exhaustivity property is unrealistic, as the news corpora commonly contain many copies of single ground truth. For example, claim “Miloš Zeman is the Czech president” can be supported using any “...”, said the Czech president Miloš Zeman.” clause occurring in corpus.

Therefore, we propose a different scheme: annotator is advised to spend 2–3 min finding as many distinct evidence sets as possible within the time needed for good reading comprehension. Furthermore, we have collected an average of 2 cross-annotations for each claim. This allowed us to merge the evidence sets across different $\textsf {T}_{\textsf {2}}$ annotations of the same claim, as well as it resulted in a high coverage of our cross-validation experiments in Sect. 4.6.1.

### Dataset analysis and postprocessing

**Table 3:** Label distribution in CTKFacts splits before and after cleaning

Split	CTKFacts uncleaned, balanced			CTKFacts cleaned, stratified		
	SUPPORTS	REFUTES	NEI	SUPPORTS	REFUTES	NEI
Train	1164	549	503	1104	556	723
Dev	100	100	100	142	85	105
Test	200	200	200	176	79	127

After completing the annotation runs, we have extracted a total of 3,116 multi-annotated claims. 47% were SUPPORTed by the majority of their annotations, REFUTES and NEI labels were approximately even, the full distribution of labels is listed in Table 3.

Of all the annotated claims, 1776, that is 57%, had at least two independent labels assigned by different annotators. This sample was given by the intrinsic randomness of $\textsf {T}_{\textsf {2}}$ claim sampling. In this section, we use it to asses the quality of our data and ambiguity of the task, as well as to propose annotation cleaning methods used to arrive to our final cleaned CTKFacts dataset.

#### Inter-annotator agreement

Due to our cross-annotation design (Sect. 4.5.3), we had generously sized sample of independently annotated labels in our hands. As the total number of annotators was greater than 2, and as we allowed missing observations, we have used the Krippendorff’s alpha measure (Krippendorff, 1970) which is the standard for this case (Hayes & Krippendorff, 2007). For the comparison with Thorne et al. (2018a) and Nørregaard and Derczynski (2021), we also list a 4-way Fleiss’ $\kappa$-agreement (Fleiss, 1971) calculated on a sample of 7.5% claims.

We have calculated the resulting Krippendorff’s alpha agreement to be 56.42% and Fleiss’ $\kappa$ to be 63%. We interpret this as an adequate result that testifies to the complexity of the task of news-based fact verification within a fixed knowledge scope. It also encourages a round of annotation cleaning experiments that would exploit the number of cross-annotated claims to remove common types of noise.

#### Manual annotation cleaning

We have dedicated a significant amount of time to manually traverse *every* conflicting pair of annotations to see if one or both violate the annotation guidelines. The idea was that this should be a common case for such annotations, as the CTK corpus does not commonly contain a conflicting pair of paragraphs except for the case of *temporal reasoning* explained in Sect. 4.7.

After separating out 14% (835) erroneously formed annotations, we have been able to resolve every conflict, ultimately achieving a full agreement between the annotations. We discuss the main noise patterns in Sect. 4.7.

#### Model-based annotation cleaning

Upon evaluating our NLI models (Sect. 6.2), we have observed that model misclassifications frequently occur at $\textsf {T}_{\textsf {2}}$ annotations that are counterintuitive for human, but easier to predict for a neural model.

Therefore, we have performed a series of experiments in model-assisted human-in-the-loop data cleaning similar to Guyon et al. (1994) in order to catch and manually purge outliers, involving an expertly trained annotator working without a time constraint:

1. A *fold* of dataset is produced using the current up-to-date annotation database, sampling a stratified test split from all untraversed claims - the rest of data is then divided into dev and train stratified splits, so that the overall train-dev-test ratio is roughly 8:1:1.
2. Mark the test claims as traversed.
3. A round of NLI models (Sect. 6.2) is trained using the current train split to obtain the strongest veracity classifier for the current fold. The individual models are optimized w.r.t. the dev split, while the strongest one is finally selected using test.
4. test-misclassifications of this model are then presented to an expert annotator along with the model suggestion and an option to remove an annotation violating the rules and to propose a new one in its place.
5. New annotations propagate into the working database and while there are untraversed claims, we proceed to step 1.

Despite allowing several inconsistencies with the scheme above during the first two folds (that were largely experimental), this led to a discovery of another 846 annotations conflicting the expert annotator’s labeling and a proposal of 463 corrective annotations (step 4).

### Common annotation problems

In this section, we give an overview of common misannotation archetypes as encountered in the cleaning stage (Sects. 4.6.2 and 4.6.3). These should be considered when designing annotation guidelines for similar tasks in the future. The following list is sorted by decreasing appearance in our data.

1. **Exclusion misassumption** is by far the most prevalent type of misannotation. The annotator wrongly assumes that an event connected to one entity implies that it cannot be connected to the other entity. E.g., evidence *“Prague opened a new cinema.”* leads to *“Prague opened a new museum.”* claim to be refuted. In reality, there is neither textual entailment between the claims, nor their negations. We attribute this error to confusing the $\textsf {T}_{\textsf {2}}$ with a *reading comprehension*^23^ task common for the field of humanities.
2. **General misannotation**: we were unable to find exact explanations for large part of the mislabelled claims. We traced the cause of this noise to both unclearly formulated claims and UI-based user errors.
3. **Reasoning errors** cover failures in assessment of the claim logic, e.g., confusing “less” for “more”, etc. Also, this often involved errors in temporal reasoning, where an annotator submits a dated evidence that contradicts the latest news w.r.t. the timestamp.
4. **Extending minimal evidence**: larger than minimal set of evidence paragraphs was selected. This type of error typically does not lead to misannotation, nevertheless, it was common in the sample of dataset we were analyzing.
5. **Insufficient evidence** where the given evidence misses vital details on entities. As an example: the evidence *“A new opera house has been opened in Copenhagen.”* does not automatically support the claim *“Denmark has a new opera house.”* if another piece of evidence connecting *Copenhagen* and *Denmark* is not available. This type of error indicates that the annotator of the claim extended the allowed *knowledge scope* with his/hers own world-knowledge.

### CTKFactsNLI dataset

Finally, we publish the resulting cleaned CTKFacts dataset consisting of 3,097 manually labeled claims and 3,716 sets of evidence for the veracity labeling, label distribution as displayed in Table 3. 727 claims have more than one valid evidence set (extracted from the independent cross-annotations described in Sect. 4.5.3). 457 evidence sets are composed of more than one document (CTK paragraph), 114 of which feature paragraphs of at least two different CTK articles.

We opt for stratified splits due to the relatively small size of our data and make sure that no CTK source paragraph was used to generate claims in two different splits, so as to avoid any *data leakage*.

The full CTK corpus cannot be, unfortunately, released publicly. Nevertheless, we extract all of our 3911 labeled *claim*-*evidence* pairs to form the CTKFactsNLI dataset. Claim-wise, it follows the same splitting as our DR dataset, and the NEI evidence is augmented by the paragraph that was used to derive the claim to enable inference experiments.

We have acquired the authorization from CTK to publish all evidence plaintexts, which we include in CTKFactsNLI and open for public usage. The dataset is released publicly on HuggingFace dataset hub^24^ and provides its standard usage API to encourage further experiments.

## Spurious cue analysis

In the claim generation phase $\textsf {T}_{\textsf {1b}}$, annotators are asked to create mutations of the initial claim. These mutations may have a different truth label than the initial claim or even be non-verifiable with the given knowledge database. During trials in Thorne et al. (2018a), the authors found that a majority of annotators had a difficulty with creating non-trivial negation mutations beyond adding “not” to the original. Similar *spurious cues* may lead to models cheating instead of performing proper semantic analysis.

In Binau and Schulte (2020), the authors investigated the impact of the trivial negations on the quality of the EnFEVER and DanFEVER datasets. Here we present similar analysis based on the *cue productivity* and *coverage* measures derived from work of Niven and Kao (2019).

In our case the cues extracted from the claims have a form of unigrams and bigrams. The definition of the *productivity* assumes a balanced dataset with respect to labels. The *productivity* of a cue *k* is calculated as follows:

1 $$\begin{aligned} \pi _k = \frac{\max \limits _{c \in C} |A_{cue = k} \cap A_{class = c}|}{|A_{cue = k}|}, \end{aligned}$$

where *C* denotes the set of possible labels $C=\{\texttt {SUPPORTS}{}, \texttt {REFUTES}{}, \texttt {NEI}{}\}$, *A* is the set of all claims, $A_{cue = k}$ is the set of claims containing cue *k* and $A_{class = c}$ is the set of claims annotated with label *c*. Based on this definition the range of *productivity* is limited to $\pi _k \in [1/|C|, 1]$, for balanced dataset. The *coverage* of a cue is defined as a ratio $\xi _{k} = |A_{cue = k}|/|A|$.

We take the same approach as Binau and Schulte (2020) to deal with the dataset imbalance: the resulting metrics are obtained by averaging over ten versions of the data based on random subsampling. We compute the metrics for both CsFEVER and CTKFacts datasets. Similarly to Binau and Schulte (2020), we also provide the harmonic mean of productivity and coverage, which reflects the overall effect of the cue on the dataset.

The results in Table 4 show that the cue bias detected in EnFEVER claims (Binau & Schulte, 2020) propagates to the translated CsFEVER, where the words “nené” (“is not”) and “pouze” (“only”) showed high productivity of 0.57 and 0.55 and ended in the first 20 cues sorted by the *harmonic mean*. However, their impact on the quality of the entire dataset is limited as their coverage is not high, which is illustrated by their absence in the top-5 most influential cues. Similar results for the CTKFacts are presented in Table 5.

**Table 4:** Cue bias (productivity, coverage and their harmonic mean) calculated on CsFEVER dataset claims sorted by the decreasing harmonic mean

Rank	Cue_cs_	Cue_translated_	Label	Productivity	Coverage	H. mean
*Unigrams*						
1	je	is	SUP	0.34	0.24	0.28
2	v	in/at	REF	0.35	0.20	0.25
3	se		REF	0.36	0.15	0.21
4	byl	was	NEI	0.37	0.09	0.14
5	na	on	NEI	0.36	0.08	0.13
*Unigram negations*						
21	není	is not	REF	0.91	0.02	0.04
61	nebyl	wasn’t	REF	0.91	0.01	0.02
114	nemá	doesn’t have	REF	0.87	0.00	0.01
171	nebyla	wasn’t	REF	0.91	0.00	0.01
294	nehrál	didn’t play	REF	0.91	0.00	0.00
*Bigrams*						
1	v roce	in year	REF	0.45	0.06	0.11
2	ve filmu	in movie	SUP	0.46	0.04	0.07
3	se narodil	was born	REF	0.46	0.02	0.04
4	Ve filmu	In movie	NEI	0.48	0.02	0.03
5	se nachází	is located	REF	0.41	0.01	0.03

**Table 5:** Cue bias (productivity, coverage and their harmonic mean) calculated on ČTK dataset claims sorted by the harmonic mean

Rank	Cue_cs_	Translation	Label	Productivity	Coverage	H. mean
*Unigrams*						
1	v	in/at	NEI	0.34	0.29	0.31
2	se		SUP	0.35	0.15	0.21
3	na	on	SUP	0.35	0.13	0.19
4	je	is	REF	0.37	0.11	0.17
5	V	In/At	NEI	0.44	0.09	0.15
*Unigram negations*						
56	není	is not	REF	0.79	0.01	0.02
96	nesouhlasí	disagrees	NEI	0.50	0.01	0.01
174	nebude	won’t	REF	0.70	0.01	0.01
218	nemí	doesn’t have	REF	0.78	0.00	0.01
696	nelegální	illegal	NEI	0.60	0.00	0.01
*Bigrams*						
1	v roce	in year	REF	0.35	0.04	0.07
2	se v	in/at	NEI	0.40	0.02	0.03
3	V roce	In year	REF	0.39	0.02	0.03
4	v Praze	in Prague	REF	0.40	0.02	0.03
5	více než	more than	SUP	0.41	0.01	0.02

## Baseline models

In this section, we explore the applicable models for both CsFEVER and CTKFacts. We train a round of currently best-performing *document retrieval* (DR) and *natural language inference* (NLI) models, to examine the difficulty of the task and to establish a baseline to our datasets, mainly CsFEVER-NLI and CTKFactsNLI. We also give results for EnFEVER providing a point of reference to the well-established dataset.

### Document retrieval

We provide four baseline models for the document retrieval stage: DrQA and Anserini represent classical keyword-search approaches, while multilingual BERT (M-Bert) and ColBert models are based on Transformer neural architectures.

In line with FEVER  (Thorne et al., 2018a), we employ document retrieval part of DrQA  (Chen et al., 2017) model. The model was originally used for answering questions based on Wikipedia corpus, which is relatively close to the task of fact-checking. The DR part itself is based on TF-IDF weighting of BoW vectors while optimized by using hashing. We calculated the TF-IDF index using DrQA implementation for all unigrams and bigrams with $2^{24}$ buckets.

Inspired by the criticism of choosing weak baselines presented in Yang et al. (2019), we decided to validate our TF-IDF baseline against the proposed Anserini toolkit implemented by Pyserini (Lin et al., 2021).

We computed the index and then finetuned the $k_1$ and *b* hyper-parameters using grid search on defined grid $k_1 \in [0.6, 1.2], b\in [0.5, 0.9],$ both with step 0.1. On a sample of 10,000 training claims, we selected the best performing parameter values: for CsFEVER these were $k_1 = 0.9$ and $b = 0.9$, while for EnFEVER and CTKFacts we proceed with $k_1 = 0.6$ and $b=0.5$.

Another model we tested is the M-Bert  (Devlin et al., 2019), which is a representative of Transformer architecture models. We used the same setup as in Chang et al. (2020) with an added linear layer consolidating the output into embedding of the required dimension 512.

In the fine-tuning phase, we used the claims and their evidence as relevant (positive) passages. For multi-hop claims,^25^ based on combinations of documents, we split the combined evidence, so the queries are always constructed to relate to a single evidence document, only. Unlike in Chang et al. (2020), we used a smaller training batch size of 128 and learning rates $10^{-5}$ for the ICT+BFS tuning and $5\times 10^{-6}$ for the fine-tuning stage.

We used this fine-tuned model to generate 512-dimensional embeddings of the whole document collection. In the retrieval phase, we used the FAISS library (Johnson et al., 2019) and constructed *PCA384 Flat* index for CTKFacts and *Flat* index for CsFEVER data.^26^

The last tested model was a recent ColBert, which provides the benefits of both cross-attention and two-tower paradigms (Khattab & Zaharia, 2020). We have employed the implementation as provided by the authors,^27^ changing the backbone model to M-Bert and adjusting for the special tokens. The training batch size was 32, learning rate $3\times 10^{-6}$, we have used masked punctuation tokens, mixed precision and L2 similarity metric.

The model was trained using triplets *(query, positive paragraph, negative paragraph)* with the objective to correctly classify paragraphs using a cross-entropy loss function. We constructed the training triplets so that the claim created by a human annotator was taken as a *query*, a paragraph containing evidence as a *positive* and a random paragraph from a randomly selected non-evidence document as a *negative* sample.

We also added triplets based on hard negatives, where instead of selecting the negative paragraphs from a random document, we picked them from an evidence document with the condition that the paragraph must not be used directly by the evidence.

As already stated, for the CTKFacts, the number of claims is significantly lower than for CsFEVER. Therefore, we have increased the overall number of CTK training triplets by generating synthetic triplets as follows. We generated a synthetic query by extracting a random sentence from a random paragraph. A set of the remaining sentences of this paragraph were designated a *positive paragraph*. The *negative paragraph* was, once again, selected as a random paragraph of an arbitrary document. Then the title was used as a query instead of a random sentence, and a random paragraph from the article was used as a *positive*. As a result, we generated about 950,000 triplets ($\approx$ 944,000 synthetic and $\approx$ 6000 using human-created claims) for the CTKFacts.

We tried two setups here, 32 and 128 dimensional term representation (denoted ColBert 32 and ColBert 128) with document trimming to a maximum of 180 tokens on FEVER datasets. The results are shown in Table 6. Methods are compared by means of Mean Reciprocal Rank (MRR) given $k\in \{1, 5, 10, 20\}$ retrieved documents. For CsFEVER, the neural network models achieve significantly best results., with ColBert taking lead. In case of CTKFacts, both Anserini and ColBert are best performers. Interestingly, M-Bert fails in this task. We found that this is mainly caused by M-Bert preference for shorter documents (including headings). As expected, the results for EnFEVER are comparable to those of CsFEVE: the Anserini performance improved, while ColBert performed slightly worse for the English corpus. Note that the EnFEVER corpus is more than ten times larger (5.4 M pages) than the CsFEVER one (452k pages).

**Table 6:** Document retrieval baseline results in MRR (%) for CsFEVER, CTKFacts, and EnFEVER datasets

dataset	model	MRR@1	MRR@5	MRR@10	MRR@20
CsFEVER	DrQA	31.23	38.55	39.14	39.38
	Anserini	27.06	32.91	33.75	34.13
	M-Bert	50.54	55.90	56.19	56.29
	ColBert 128	**63**.**93**	**70**.**70**	**71**.**16**	**71**.**34**
CTKFacts	DrQA	9.26	14.25	15.28	15.55
	Anserini	13.23	**18**.**65**	**19**.**39**	**19**.**78**
	M-Bert	1.59	2.39	2.75	3.18
	ColBert 32	**13**.**49**	18.50	19.26	19.70
EnFEVER	DrQA	27.09	36.06	37.51	38.08
	Anserini	32.87	41.48	42.44	42.91
	ColBert 128	**53**.**57**	**64**.**03**	**64**.**73**	**64**.**94**

Bold font signifies the best-performing model in each experiment

### Natural language inference

The aim of the final stage of the fact-checking pipeline, the NLI task, is to classify the veracity of a claim based on textual evidence.^28^ We have fine-tuned several different Transformer models trained on Czech language tasks on our data in order to provide a strong NLI baseline on our datasets.

From the multilingual models, we have experimented with SlavicBERT and Sentence M-Bert  (Reimers & Gurevych, 2019) models in their *cased* defaults, provided by the DeepPavlov library (Burtsev et al., 2018), as well as with the original M-Bert from Devlin et al. (2019), Pires et al. (2019).

We have further examined two pretrained XLM-RoBERTa-large models, one fine-tuned on an NLI-related *SQuAD2* (Rajpurkar et al., 2016) *down-stream task*, other on the crosslingual *XNLI* (Conneau et al., 2018) task. These were provided by Deepset^29^ and HuggingFace^30^

Finally, we have performed a round of experiments with a pair of recently published Czech monolingual models. RobeCzech  (Straka et al., 2021) was pretrained on a set of currated Czech corpora using the RoBERTa-base architecture. FERNET-C5  (Lehečka & Švec, 2021) was pretrained on a large crawled dataset, using Bert-base architecture.

We have fine-tuned these models using 4 fact-checking datasets adapted for the NLI task using their gold evidence sets:

- CTKFactsNLI uses our data collected in Chapter 4, extracting them into pairs of strings—claim and its evidence (or the text of its *source paragraph* for NEI claims)—and their respective veracity labels.
- FEVER-NLI was extracted from the original EnFEVER dataset in Nie et al. (2019), emulating the missing NEI evidence by a sample of 3–5 results of the proposed DR model.
- CsFEVER is a Czech translation of FEVER-NLI (see Sect. 3.3).
- CsFEVER (*NearestP*) was obtained from the CsFEVER dataset introduced in Sect. 3 using the entire Wiki abstracts for evidence. Non-verifiable claim evidence was chosen using the *nearest page* method (Thorne et al., 2018a)—picking the top result of our DrQA model established in Sect. 6.1.

For each dataset, we have fine-tuned all listed models using the sentence_transformers implementation of the Cross-encoder (Reimers & Gurevych, 2019) with two texts on input (evidence, claim) and a single output value (SUPPORTS/REFUTES/NEI). We have experimented with multiple batch sizes for each model (varying from 2 to 10) and trained each using the Adam optimizer with $2 \times 10^{-5}$ default learning rate and weight decay of 0.01. The number of linear warmup steps was determined by 10–30% of the train size, varying per experiment. Ultimately, we kept the best performing models in terms of dev accuracy for each model-dataset pair.

We then evaluated all models using the test-splits of the dataset they were fine-tuned with. Results are presented in Table 7 and show dominance of the XLM-RoBERTa -large models fine-tuned on a related multilingual task and then fine-tuned again using the train split of the examined dataset. For reference, we also compare our results with previous research on English datasets—in case of FEVER-NLI (and its Czech translation), our models achieved superiority over the NSMNs published in Nie et al. (2019) which scored an overall 69.5 macro-F1. Our baselines for CTKFactsNLI and CsFEVER (*NearestP*) achieved and F-score of 76.9 and 83.2%, respectively. This is comparable with the Thorne et al. (2018a) baseline which scored 80.82% *accuracy* in a sentence-level *NearestP* setting on EnFEVER, using the Decomposable Attention model. Interestingly, the CsFEVER (*NearestP*) experiment results are consistently higher than those of CsFEVER-NLI—we consider this estimate overly optimistic and attribute it to a possible partial information leakage discussed in Sect. 3.1 and the CsFEVER noise examined in Sect. 3.2.1.

**Table 7:**
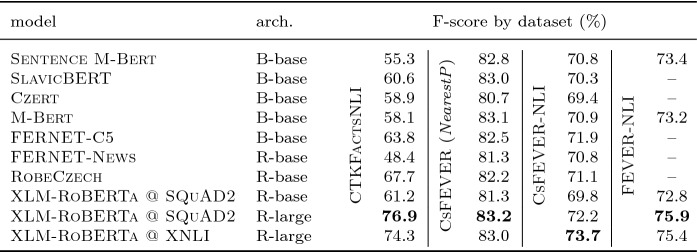
F1 macro score (%) comparison of our Bert-like models fine-tuned for the *NLI* task on CTKFactsNLI, CsFEVER, and CsFEVER-NLI datasets

Bold font signifies the best-performing model in each experiment

Gold evidence was used as the NLI context for each claim. FEVER-NLI from Nie et al. (2019) listed for comparison with other research. B stands for Bert architecture, R for RoBERTa

### Full pipeline results

Similarly to Thorne et al. (2018a), we give baseline results for the full fact verification pipeline. The pipeline is evaluated as follows: 1) given a mutated claim *m* from the test set, *k* evidence paragraphs (documents) $P = \{p_1, \ldots , p_k\}$ are selected using document retrieval models as described in Sect. 6.1. Note that documents in *P* are ordered by decreasing relevancy. The paragraphs are subsequently fed to an NLI model of choice (see details below), and accuracy (for CsFEVERand EnFEVER) or F1 macro score (for the unbalanced CTKFacts) are evaluated. In case of supported and refuted claims, we analyze two cases: (1) for Score Evidence (SE), *P* must fully cover at least one gold evidence set, (2) for No Score Evidence (NSE) no such condition applies. No condition applies for NEI claims as well.^31^

While our paragraph-oriented pipeline eliminates the need for sentence selection, we have to deal with the maximum input size of the NLI models (512 tokens in all cases), which gets easily exceeded for larger *k*. Our approach is to iteratively partition *P* into *n* consecutive splits $S = \{s_1, \ldots , s_l\}$, where $l \le n$. Each split $s_i$ itself is a concatenation of successive documents $s_i = \{p_s, \ldots , p_e\}$, where $1\le s \le e\le n$. A new split is created for any new paragraph that would cause input overflow. If any single tokenized evidence document is longer than the maximum input length, it gets represented by a single split and truncated.^32^ Moreover, each split is limited to at most $k_s$ successive evidence documents ($k_s=2$ for CsFEVER and EnFEVER, $k_s=3$ for CTKFacts), so the overall average input length is more akin to data used to train the NLI models.

In the prediction phase, all split documents $p_s, \ldots , p_e$ are concatenated, and, together with the claim *m*, fed to the NLI model getting predictions $y_s, \ldots , y_e$, where each $y_i = (y_i^{\texttt {SUPPORTS}{}}, y_i^{\texttt {REFUTES}{}}, y_i^{\texttt {NEI}{}})$ represents classification confidences. Finally, the claim-level confidences are obtained as $y^c = \frac{1}{e-s}\sum _{i=0}^{e-s}\lambda ^i y_i^c$ for $c \in \{\texttt {SUPPORTS}{}, \texttt {REFUTES}{}, \texttt {NEI}{}\}$. This weighted average (we use $\lambda =\frac{1}{2}$ in all cases) assigns higher importance to the higher-ranked documents.

The results are presented in Table 8. We evaluate Anserini and ColBert DR models followed by overall best performing XLM-RoBERTa @ SQuAD2 NLI model (RoBERTa-large, described in Sect. 6.2) for all datasets. For both FEVER-based datasets, ColBert document retrieval brings significantly the best results. For CTKFacts, Anserini and ColBert perform similarly with Anserini giving slightly better results overall which mimics the results of DR described in Sect. 6.1. Note that the SE to NSE difference is more pronounced for CTKFacts, which can be explained by high redundancy of CTKFacts paragraphs w.r.t. CsFEVER. Comparing the results of the EnFEVER baseline to Thorne et al. (2018a) our models perform better in spite of discarding the sentence selection stage—the best reported EnFEVER accuracies for 5 retrieved documents were $52.09\%$ for NSE and $32.57\%$ for SE (Thorne et al., 2018a). The improvement can be explained by more recent and sophisticated models used in our study [authors of Thorne et al. (2018a) used Decomposable Attention (Parikh et al., 2016) at the NLI stage]. Note that EnFEVER full pipeline significantly outperforms CsFEVER, which we explain by the information leakage and noise of the dataset used to train the CsFEVER (*NearestP*) model as discussed in the previous section.

**Table 8:** Full pipeline results

Dataset	Retrieval	@1		@5		@10		@20	
		NSE	SE	NSE	SE	NSE	SE	NSE	SE
CsFEVER	Anserini	49.13	14.17	50.59	18.69	50.65	19.89	50.67	20.65
	ColBert 128	**61**.**90**	*25.67*	*58.89*	*29.81*	*59.03*	*30.31*	*59.23*	**30**.**37**
CTKFacts	Anserini	*60.06*	*11.24*	*58.35*	*19.97*	**59**.**69**	*24.79*	*59.50*	**26**.**71**
	ColBert 32	59.94	10.68	56.97	19.28	57.23	22.74	56.39	25.51
EnFEVER	Anserini	66.47	22.44	65.72	34.10	65.59	35.97	65.37	35.39
	ColBert 128	**68**.**19**	*33.83*	*66.21*	*46.15*	*65.73*	*48.20*	*65.43*	**49**.**16**

For each dataset, bold font signifies the best result in each experiment setting, italics signify the best result for each retrieval size and setting

Accuracy (%) shown for CsFEVER, and EnFEVER, F1 macro score (%) for CTKFacts. Unlike NSE (No Score Evidence), SE (Score Evidence) demands correct evidence to be retrieved

## Conclusion

With this article, we examined two major ways to acquire Czech data for automated fact-checking.

Firstly, we localized the EnFEVER dataset, using a document alignment between Czech and English Wikipedia abstracts extracted from the *interlingual links*. We obtain and publish the CsFEVER dataset of 127k machine-translated claims with evidence enclosed within the Czech Wikipedia dump. We then validate our alignment scheme and measure a 66% precision using hand annotations over a 1% sample of obtained data. Therefore, we recommend the data for models less sensitive to noise and we proceed to utilize CsFEVER for training non-critical retrieval models for our annotation experiments and for recall estimation of our baseline models. Furthermore, we publish a CsFEVER-NLI dataset of 228k context-query pairs directly translated from English to Czech that bypass its issue with noise for the subroutine task of Natural Language Inference.

Secondly, we executed a series of human annotation runs with 163 students of journalism to acquire a novel dataset in Czech. As opposed to similar annotations that extracted claims and evidence from Wikipedia  (Thorne et al., 2018a; Nørregaard & Derczynski, 2021; Aly et al., 2021), we annotated our dataset on top of a CTK corpus extracted from a news agency archive to explore this different relevant language form. We collected a raw dataset of 3,116 labeled claims, 57% of which have at least two independent cross-annotations. From these, we calculate Krippendorff’s alpha to be 56.42% and 4-ways Fleiss’ $\kappa$ to be 63%. We proceed with manual and human-and-model-in-the-loop annotation cleaning to remove conflicting and malformed annotations, arriving at the thoroughly cleaned CTKFacts dataset of 3,097 claims and their veracity annotations complemented with evidence from the CTK corpus. We release its version for NLI called CTKFactsNLI to maintain corpus trade secrecy.

Finally, we use our datasets to train baseline models for the full fact-checking pipeline composed of Document Retrieval and Natural Language Inference tasks.

### Future work

- The fact-checking pipeline is to be augmented by the *check-worthiness estimation* (Nakov et al., 2021), that is, a model that classifies which sentences of a given text in Czech are appropriate for the fact verification. We are currently working on models that detect claims within the Czech Twitter, and a strong predictor for this task would also strengthen our annotation scheme from Sect. 4.5 that currently relies on hand-picked check-worthy documents.
- While the SUPPORTS, REFUTES and NEI classes offer a finer classification w.r.t. evidence than binary true/false, it is a good convention of fact-checking services to use additional labels such as MISINTERPRETED, that could be integrated into the common automated fact verification scheme if well formalised.
- The claim extraction schemes like that from Thorne et al. (2018a) or Sect. 4.5 do not necessarily produce organic claims capturing the real-world complexity of fact-checking. For example, just the EnFEVER train set contains hundreds of claims of form “X is a person.”. This problem does not have a trivial solution, but we suggest integrating real-world claims sources, such as Twitter, into the annotation scheme.
- While the FEVER localization scheme from Sect. 3.1 yielded a rather noisy dataset, its size and document precision encourage deployment of a model-based cleaning scheme like that from Jeatrakul et al. (2010) to further refine its results. I.e., a well performing NLI model could do well in pruning the invalid datapoints of CsFEVER without further annotations.

## Figures and Tables

**Fig. 3:**
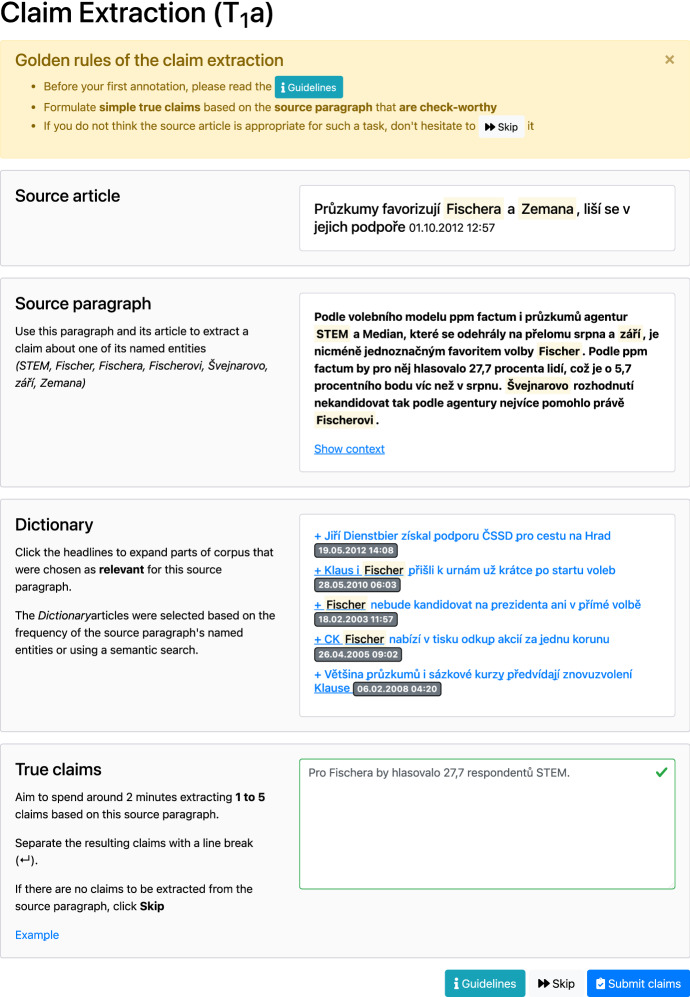
Claim extraction interface of the FCheck platform. Captions were translated to English, corpus data kept in Czech

**Fig. 4:**
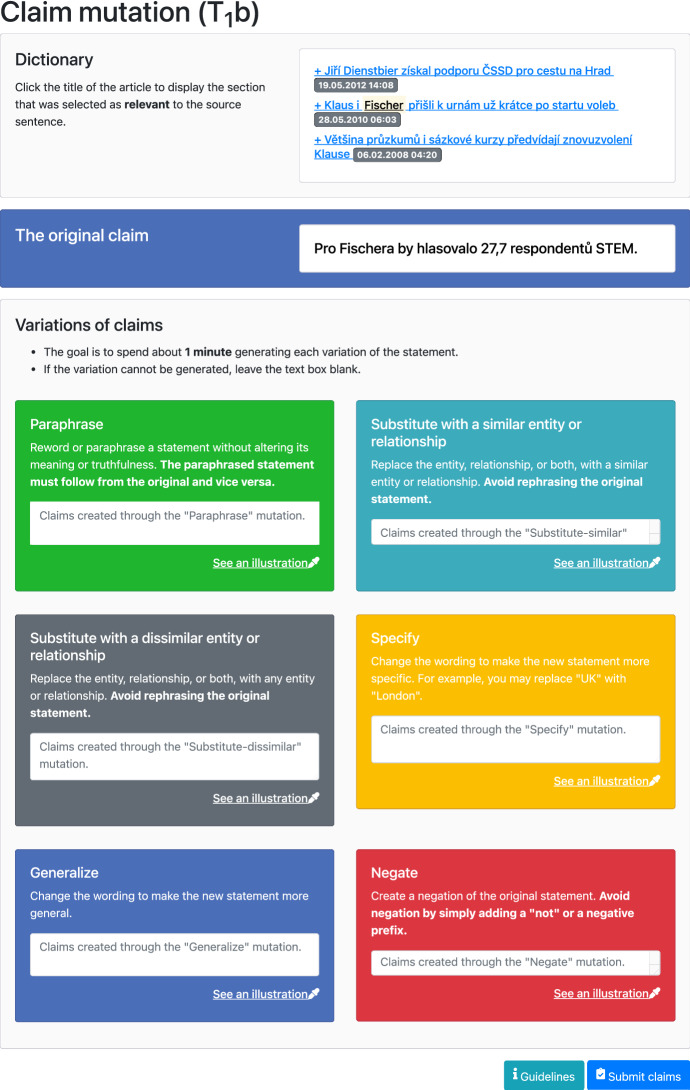
Claim mutation interface. Captions were translated to English, corpus data kept in Czech

**Fig. 5:**
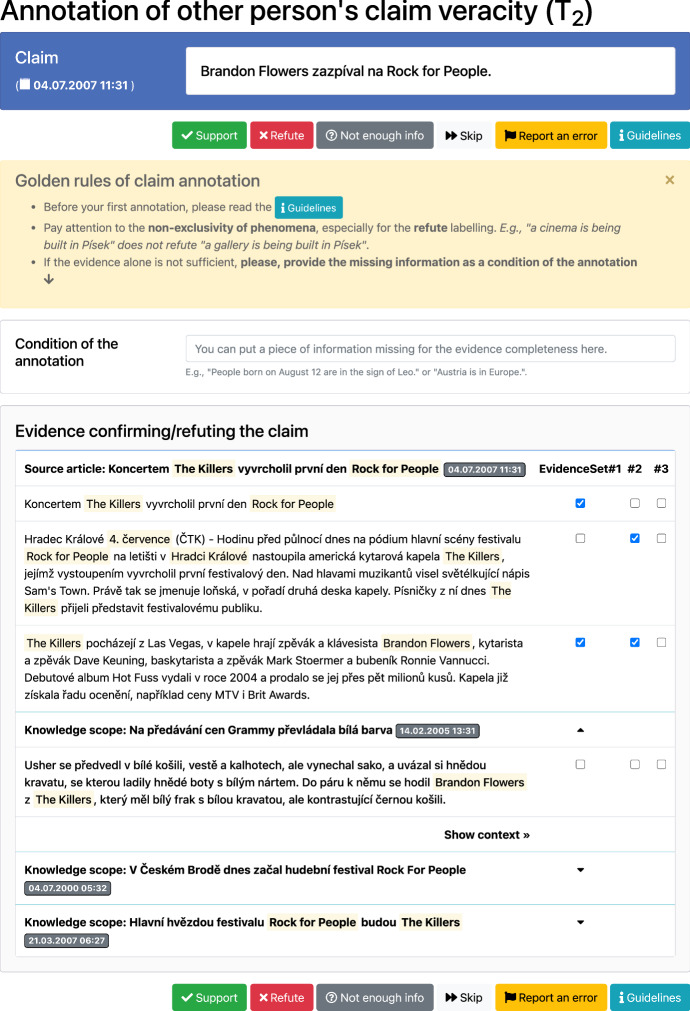
Claim labelling interface. Captions were translated to English, corpus data kept in Czech

## Appendix 1: Annotation platform

### Claim extraction

See Figs. 3 and 4.

Figure 5 shows the *Claim Extraction* ($\textsf{T}_{\textsf {1a}}$) interface.

The layout is inspired by the work of Thorne et al. (2018a) and, by default, hides as much of the clutter away from the user as possible. Except for the article heading, timestamp, and source paragraph, all supporting information is collapsed and only rendered on user demand.

An annotator reads the source article and, if it lacks a piece of information he/she wants to extract, looks for it in the expanded article or corpus entry. The user is encouraged to Skip any source paragraph that is hard to extract—the purpose of *Source Document Preselection* ($\textsf {T}_{\textsf {0}}$) was to reduce these skips as much as possible.

Throughout the platform, we have ultimately decided not to display any *stopwatch*-like interface not to stress out the user. We have measured that, excluding the outliers ($\le \,10$ s, typically the Skipped annotations, and $\ge \, 600$ s, typically a browser tab left unattended), average time spent on this task is **2 minutes 16 seconds** and the median is **1 minutes 16 seconds**.

### Claim mutation

Mutation types follow those of the FEVER Annotation Platform and are distinguished by loud colors, to avoid mismatches.

Excluding the outliers, the overall average time spent generating a batch of mutations was **3m 35s** (median **3m 15s**) with an average of **3.98** mutations generated per claim.

### Claim veracity labeling

In Fig. 5 we show the most complex interface of our platform—the $\textsf {T}_{\textsf {2}}$: **Claim Annotation** form. Full instructions took about 5 min to read and comprehend.

The input of multiple evidence sets works as follows: each column of checkboxes in Fig. 5 stands for a single evidence set, every paragraph from the union of knowledge belongs to this set iff its checkbox in the corresponding column is checked. Offered articles and paragraphs are collapsible, empty evidence set is omitted.

On average, the labelling task took **65 seconds**, with a median of **40 seconds**. An average SUPPORTS/REFUTES annotation was submitted along with **1**.**29** different evidence sets, 95% of which were composed of a single paragraph.

## Footnotes

1 https://factcheck.afp.com/.

2 https://www.poynter.org/ifcn/.

3 https://www.politifact.com/.

4 https://www.poynter.org/.

5 https://www.snopes.com/.

6 https://www.claimreviewproject.com/.

7 https://www.crowdtangle.com/.

8 And the veracity of such claim may be further nuanced by the affiliation and bias of the speaker.

9 https://www.ctk.eu/.

10 https://demagog.cz/.

11 https://www.mediawiki.org/.

12 https://www.mediawiki.org/wiki/API:REST_API.

13 https://github.com/attardi/wikiextractor.

14 Estimated using MorphoDiTa (Straková et al., 2014).

15 https://cloud.google.com/translate.

16 https://deepl.com/.

17 https://huggingface.co/datasets/ctu-aic/csfever.

18 https://www.ctk.eu/.

19 See Czech News Agency codex at https://www.ctk.cz/o_ctk/kodex/ (Czech only).

20 Headline being considered as the 0-th paragraph.

21 Claim or paragraph formed in natural language accompanied by a timestamp of a date at which the knowledge should hold.

22 https://fcheck.fel.cvut.cz.

23 The corresponding reading comprehension task might be: *“Does the article tell us that Prague opened a museum? Highlight the relevant information.”*

24 https://huggingface.co/datasets/ctu-aic/ctkfacts_nli.

25 Multi-hop claim’s evidence set contains more than one document.

26 We do not provide M-Bert results for EnFEVER due to computational complexity and better results of ColBert demonstrated for CsFEVER and CTKFacts (see below).

27 https://github.com/stanford-futuredata/ColBERT.

28 While the evidence may consist of multiple strings in our fact-checking paradigm, we are always considering their concatenation—a single string—in this section.

29 https://www.deepset.ai.

30 https://huggingface.co.

31 Our approach corresponds to the NearestP strategy from Thorne et al. (2018a). Similarly, SE and NSE correspond to ScoreEv and NoScoreEv.

32 This is the only case in which truncation occurs.
